# CNVs-microRNAs Interactions Demonstrate Unique Characteristics in the Human Genome. An Interspecies *in silico* Analysis

**DOI:** 10.1371/journal.pone.0081204

**Published:** 2013-12-02

**Authors:** Harsh Dweep, George D. Georgiou, Norbert Gretz, Constantinos Deltas, Konstantinos Voskarides, Kyriacos Felekkis

**Affiliations:** 1 Medical Research Center, University of Heidelberg, Mannheim, Germany; 2 Department of Life and Health Sciences, University of Nicosia, Nicosia, Cyprus; 3 Molecular Medicine Research Center and Department of Biological Sciences, University of Cyprus, Nicosia, Cyprus; 4 St. George's University of London Medical School at the University of Nicosia, Nicosia, Cyprus; University of Torino, Italy

## Abstract

MicroRNAs (miRNAs) and copy number variations (CNVs) represent two classes of newly discovered genomic elements that were shown to contribute to genome plasticity and evolution. Recent studies demonstrated that miRNAs and CNVs must have co-evolved and interacted in an attempt to maintain the balance of the dosage sensitive genes and at the same time increase the diversity of dosage non-sensitive genes, contributing to species evolution. It has been previously demonstrated that both the number of miRNAs that target genes found in CNV regions as well as the number of miRNA binding sites are significantly higher than those of genes found in non-CNV regions. These findings raise the possibility that miRNAs may have been created under evolutionary pressure, as a mechanism for increasing the tolerance to genome plasticity. In the current study, we aimed in exploring the differences of miRNAs-CNV functional interactions between human and seven others species. By performing *in silico* whole genome analysis in eight different species (human, chimpanzee, macaque, mouse, rat, chicken, dog and cow), we demonstrate that miRNAs targeting genes located within CNV regions in humans have special functional characteristics that provide an insight into the differences between humans and other species.

## Introduction

MicroRNAs (miRNAs) constitute a class of short endogenous non-coding RNA molecules of 21–25 nucleotides (nt) in length which function as negative regulators of gene expression at the post-transcriptional level in multicellular eukaryotes [1]. We have recently studied the possible interfering role of miRNAs in relation with Copy Number Variations (CNVs). CNVs is a newly discovered category of genomic elements which encompass segmental duplications of greater than 1 kb, and frequently include protein encoding genes[2]. By performing *in silico* whole genome analysis, it has been demonstrated that both the number of miRNAs that target genes found in CNVs regions as well as the number of miRNA binding sites are significantly higher than those of genes found in non-CNV regions[3], [4]. These findings raise the possibility that miRNAs may have been created under evolutionary pressure, as a mechanism for increasing the tolerance to genome plasticity. In the current study, our main hypothesis is that this particular function of miRNAs and their close relation with CNVs may be more significant in human lineage due to evolution of special characteristics in human physiology.

Previous studies attempted to detect similarities and differences between human and other species, especially in comparison with other primates, regarding miRNAs and CNVs. Brameier *et al*. (2011), found that only 40 percent of other primates miRNAs are identical with their human ortholog[5]. On the other hand, there is substantial evidence that miRNAs may have played an important role in the evolution of human brain through expression regulation of neuron specific coding genes[6], [7]. Gazave *et al*., performed comparative genomic hybridization in the four great apes species and suggested that CNVs are subject to different selective pressures in different lineages[8]. Additionally, Gokcumen *et al*., detected 34 hotspot regions of CNV formation that are common in human, chimpanzee and macaque genomes [9].

Despite this progress, a direct comparison of the interactions of CNVs-miRNAs across different species has not been performed yet. In theory, however, one expects that certain cellular pathways in the human lineage may have evolved under the strict requirement for close regulation or buffering in order to accommodate increased gene expression as a result of segmental duplications.

Here, we conducted a comparative *in silico* analysis for miRNA interactions on genes found in CNV regions. This comparative analysis included the genomes of 8 different species (human, chimpanzee, macaque, mouse, rat, chicken, dog and cow) and encompassed only genes that are human homologues.

It was predicted that miRNAs regulate more closely the CNV genes as well as the specific pathways in humans, as compared with the other species.

Analysis of the CNV genes that are predicted to be targets of these miRNAs revealed a special regulation of particular signaling pathways in humans that is absent in other lineages. In addition, miRNAs hosted within human CNV genes seem to have a special evolutionary role, a role that probably is related to some human disorders such as cancer. These findings reveal once again that gene expression regulation has special characteristics in the human lineage.

## Results

### Acquisition of homologous CNV genes and prediction datasets

In order to explore the nature of the miRNA-CNV interactions in different species, human CNV genes were downloaded from the DGV database. For the other seven species, the corresponding homologous genes that are homologous to the human genes were acquired from the HomoloGene database. Thereafter, a comprehensive atlas of homologous CNV and non-CNV genes was constructed by mapping the human CNV genes with the genes of 7 other species. A total of 9,337, 8,639, 9,543, 8,778, 7,139, 9,161 and 9,067 homologous CNV genes were observed in chimp, macaque, mouse, rat, chicken, dog and cow genomes, respectively. **Table 1** describes the number of CNV and non-CNV genes within 8 different species.

**Table 1:** Overview of the distribution of human homologous genes within CNV and non-CNV regions of 8 different species.

Species	CNV (N)	non-CNV (N)
**Human**	10,529	8,080
**Chimpanzee**	9,397	7,391
**Macaque**	8,639	9,796
**Mouse**	9,543	7,248
**Rat**	8,778	6,761
**Chicken**	7,139	5,241
**Dog**	9,161	7,051
**Cow**	9,067	7,113

**Figure 1:**
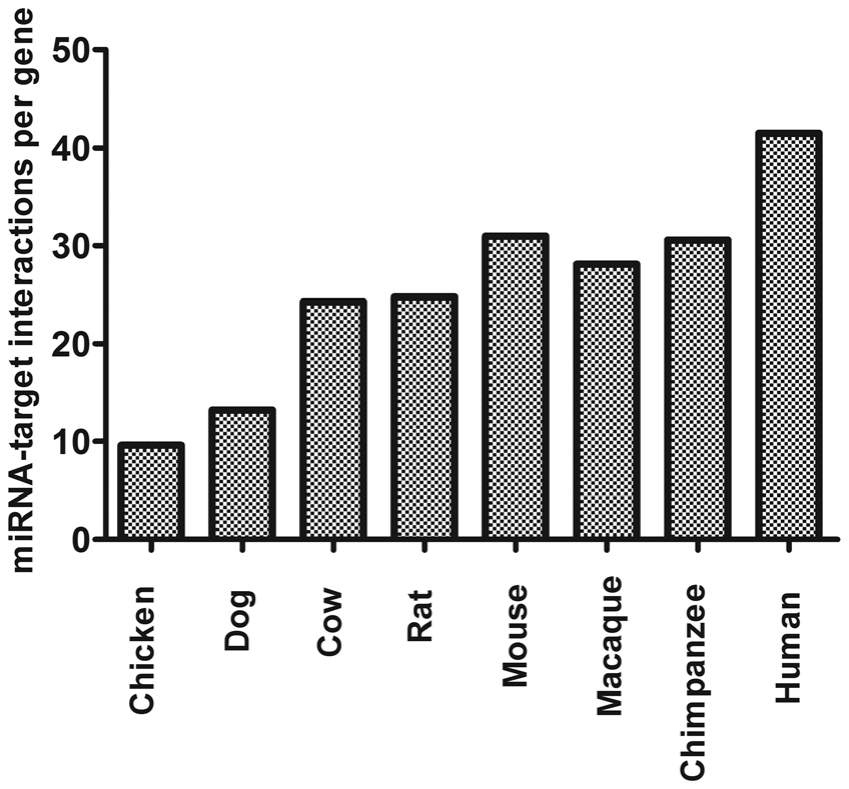
miRNA-target interactions per gene in CNV genes among the 8 different species (human, chimpanzee, macaque, mouse, rat, cow, dog, and chicken). Values represent the number of miRNA-CNV interactions, normalized by the total number of CNV genes in each species.

The putative miRNA binding site interactions within the CNV genes of 8 different species were predicted by adapting two different algorithms: miRWalk and Targetscan. These two algorithms are chosen due to their popularity as well as to reduce the number of false positive interactions. Therefore, only the miRNA-target interaction pairs predicted by both algorithms were selected for further analysis. We ended up with 436,972 (n = 10,529), 287,160 (n = 9,397), 242,653 (n = 8,639), 295,477 (n = 9,543), 217,346 (n = 8,778), 68,653 (n = 7,139), 120,852 (n = 9,161) and 219,960 (n = 9,067) miRNA-target interactions, whereas 344,830, 225,672, 193,136, 238,554, 176,696, 51,728, 97,397, 177,353 miRNA family-target pairs were estimated within the CNV genes of human, chimpanzee, macaque, mouse, rat, chicken, dog and cow, respectively. On comparing, the miRNA binding sites per gene among the 8 different species demonstrated that the human CNV genes are targeted by more miRNAs, based on the predictions of the two algorithms (**Figure 1**). These observations confirm our previous results in which it was demonstrated that both the number of miRNAs that target genes found in CNV regions as well as the number of miRNA binding sites are highly significantly enriched in humans. Collectively, these results suggest that human CNV genes are under more selective pressure of miRNA-mediated regulation relative to the 7 other species. In order to exclude that this phenomenon is attributed to the difference of the 3′-UTR length in the different species, the normalized mean length of the 3′-UTR among the 8 different species was compared. As expected the normalized means of 3′-UTR lengths of CNV genes of 8 species were not found to be significantly different (data not shown).

### Pathway enrichment analysis of CNV genes of 8 species

We attempted to derive the functional impact of the possible miRNA-CNV genes interaction pairs within the biological pathways. To achieve this task, the average means of all known miRNAs and their families were calculated by counting the total number of predicted targets per miRNA and their families. Thereafter, an overall mean for miRNA and family was estimated by using average means for 8 species. The overall miRNAs means for human, chimpanzee, macaque, mouse, rat, cow, dog and chicken, and were 532.89, 350.19, 42.06, 53.36, 36.5, 10.03, 20.55, and 38.93, respectively, whereas, 692.61, 447.2, 55.1, 73.8, 50.35, 12.3, 52.87, and 27.93 were applied as overall miRNA family means, respectively. The predicted CNV genes were then classified into two lists i.e. above and below the aforementioned means with a defined cut-offs. These lists were then interrogated for the identification of significantly enriched pathways.

**Figure 2:**
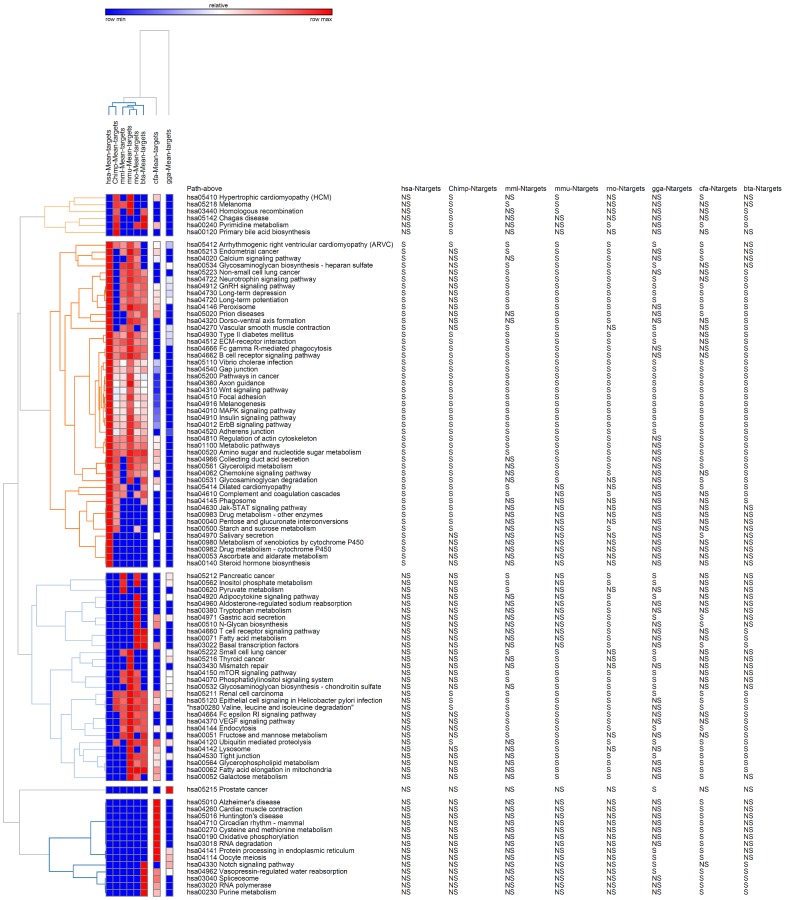
Pathway heat-map of CNV genes above the overall miRNA mean average in human (hsa), chimpanzee (chimp), macaque (mml), mouse (mmu), rat (rno), cow (bta), dog (cfa) and chicken (gga).

We determined 47 and 45 significant pathways on above and below overall mean cut-offs for miRNA-CNV interactions, whereas, 46 significant pathways were found in both categories for miRNA families-CNV interactions within human CNV genes. **Table 2** depicts an overview of significant pathways obtained on miRNAs and their family within 8 species. **Figure 2** shows the heat map of significant pathways above overall mean cut-offs within 8 different species. In general terms it can be considered that the significantly enriched pathways identified on the CNV genes (belonging to above the overall mean cut-off) are those signaling pathways which receive a higher degree of miRNA regulation.

**Table 2:** Overview of significantly enriched pathways obtained on above and below average mean cut-offs on 8 species.

Cut-off	Human	Chimpanzee	Macaque	Mouse	Rat	Cow	Dog	Chicken
**miRNA-above-pathways**	47	40	44	57	60	61	55	38
**miRNA-below-pathways**	45	44	43	57	58	61	53	40
**miRFam-above-pathways**	46	43	46	63	49	62	53	36
**miRFam-below-pathways**	46	46	39	55	53	58	51	35

Several key pathways appear to be human and chimpanzee specific encompassing various metabolic pathways such as drug metabolism, starch and sucrose metabolisms, pentose and glucose interconversion. More importantly, the human specific significant pathways also involve cellular metabolic activities. These pathways include metabolism of xenobiotics, drug metabolism, steroid hormone biosynthesis and ascorbate and aldarate metabolism (**Figure 2**). On the other hand, certain pathways associated with human diseases such as renal cell carcinoma, pancreatic cancer, signaling in Helicobacter pylori infection are predicted to lose tight miRNA regulation in humans (**Figure 2**).

### Classification of homologous genes into curated gene classes

The homologous genes (CNV and non-CNV) were mapped and separated into curated gene classes. **Table 3** depicts the number of CNV and non-CNV genes categorized under the various gene classes in all eight species. Notably, a large number of genes which encode for cell differentiation, homeodomain, oncogene, kinases, transcription factors and translocated-cancer proteins classes were found under the CNVs. Interestingly, the class of transcription factors was found to have the maximum number of CNV genes in 8 species.

**Table 3:** Classification of homologous genes (CNV/non-CNV) into the curated gene classes.

Genes/Curated classes	Cell differentiation	Cytokines and growth factors	Homeodomain	Oncogene	Kinases	Transcription factors	Translocated cancer genes	Tumor suppressor genes
**Human (CNV/non-CNV)**	200/154	215/219	143/87	212/114	329/185	833/578	189/99	44/42
**Chimpanzee (CNV/non-CNV)**	183/144	193/205	126/81	193/103	299/174	739/525	173/89	43/31
**Macaque (CNV/non-CNV)**	166/129	180/195	105/76	172/101	278/154	670/493	152/89	38/36
**Mouse (CNV/non-CNV)**	162/139	183/195	133/84	198/109	321/179	759/537	176/94	43/37
**Rat (CNV/non-CNV)**	153/132	177/184	120/76	179/102	300/167	679/478	158/89	42/37
**Chicken (CNV/non-CNV)**	106/83	104/109	102/66	173/97	276/146	563/387	153/83	34/34
**Dog (CNV/non-CNV)**	158/136	168/181	117/78	197/99	314/180	727/507	175/86	41/37
**Cow (CNV/non-CNV)**	150/134	173/198	121/81	194/106	309/175	712/529	172/91	41/38

### Genomic location scanning of homologous genes of 8 species

It has previously been described that a larger number of mammalian miRNA genes are co-expressed with their host or neighbouring genes and control the expression of host genes by exerting synergistic, and/or antagonistic regulatory effect [10]. To determine such regulatory circuits within CNV and non-CNV genes, the genomic coordinates of homologous genes and known miRNAs of 8 species were downloaded from NCBI and miRBase release 18, respectively. Afterwards, the genomic locations of homologous genes were scanned against the coordinates of known miRNAs using a customized “*Perl script*”. Interestingly, most gemomes were noted to have more miRNAs within CNV genes instead of non-CNV locations, with the exception of chimpanzee, macaque and rat genomes that have similar numbers of miRNAs in both (CNV and non-CNV genes). These findings indicate that the expression of CNV genes in some species may be controlled by miRNA-mediated synergistic and/or antagonistic regulatory effects. However, further work is required to elucidate the contribution of “host” miRNAs in the regulation of CNV genes. Table 4 illustrates a distribution map of known miRNAs within CNV and non-CNV genes among 8 different species.

**Table 4:** Distribution of known miRNA within CNV and non-CNV genes among 8 different species.

Type	Human	Chimpanzee	Macaque	Mouse	Rat	Chicken	Dog	Cow
**CNV (miRNAs/Genes)**	289/10529	75/9397	49/8639	200/9543	41/8778	83/7139	47/9161	112/9067
**non-CNV (miRNAs/Genes)**	191/8080	73/7391	55/9796	64/7248	39/6761	31/5241	7/7057	71/7113

### Overrepresentation analysis of miRNA binding sites within significant pathways and curated gene classes

An overrepresented analysis was conducted to examine the regulatory effect of miRNA binding sites within significant pathways and curated classes that were detected within human CNV genes. As shown in **Table 5**, six pathways (cancer, focal adhesion, glycerolipid metabolism, Mapk, GnRH signaling and neurotophin) were observed as significantly enriched for binding sites of miRNAs that come under the above mean cut-off category. At the same time, the same pathways as well as an additional signaling (calcium signaling) were observed as significantly enriched for the binding sites of miRNAs that belong to the below mean cut-off class within human CNV genes. Notably, the “pathways in cancer” was also determined as the highly significantly enriched pathway for miRNA binding sites for both categories (the above and the below mean cut-off).

**Table 5:** Overview of the significant pathways found enriched for the binding sites of miRNAs within human CNV genes.

Pathways	miRNA-above	miRNA-below
**Pathways in cancer**	0.01	0.009
**Focal adhesion**	0.03	0.03
**Glycerolipid metabolism**	0.04	0.03
**MAPK**	0.04	0.03
**GnRH signaling**	0.05	0.03
**Neurotrophin**	0.05	0.05
**Calcium signaling**	NS	0.03

Values indicate p values for each specific pathway.

Moreover, four curated gene classes (oncogene, transcription factors, translocated cancer genes and kinases), were also identified as significantly enriched for miRNA binding sites, whereas, the remaining gene classes were detected as insignificant (**Table 6**). This classification agrees with the pathway enrichment analysis - as the genes belonging to these four classes are known to participate in cancer development and progression in humans.

**Table 6:** Overview of miRNA binding sites enrichment analysis within the different gene classes of human CNV genes.

Classes	miRNAs	miRfams
**Cell Differentitation**	NS	NS
**Cytokin & Growthfactor**	NS	NS
**Homeodomain**	NS	NS
**miRNAProcessingProtein**	NS	NS
**Oncogene**	0.01	NS
**Transcription factors**	0.007	NS
**Translocated cancer genes**	0.01	NS
**Tumour suppressor genes**	NS	NS
**Protein Kinases**	0.01	NS

## Discussion

miRNAs are relatively novel molecules, but their role in evolution has been extensively studied. It is of considerable interest that the number of miRNAs in the genome was shown to correlate with the morphological complexity of the organism indicating that they play a role in the evolutionary change of the body structure. It is now widely accepted that an increase in the complexity of the gene expression regulatory mechanisms will drive the evolution of more complex organisms. The molecular networks interconnecting miRNAs with other genomic elements contribute significantly to such evolutionary processes.

CNVs and miRNA interactions appear to have an important evolutionary implication. It has been already shown that genes found in CNV regions are evolutionarily subjected to higher miRNA regulation as such genes are predicted to be regulated by more miRNAs and harbor more abundant miRNA binding sites compared with their non-CNV counterparts [3], [4]. A crucial question, however, is whether these CNV-miRNA interactions are unique in human linage due to evolution of special characteristics in human physiology. In this *in silico* analysis of the CNV-miRNA interactions within eight different species, we demonstrate that human CNV genes are more tidily regulated by miRNAs than CNV genes in other lineages. In addition, pathway overrepresentation analysis reveals that miRNA regulation of gene expression in the human lineage extents to unique pathways as compared with other lineages.

The classification of CNV-miRNA interactions in the eight different species into above and below- mean averages reveals a specific functional implication of the miRNA dependent regulation in humans. Such analysis highlights a variety of cellular pathways that appear to be uniquely regulated by miRNAs in humans. Interestingly, most of these pathways seem to regulate cellular metabolic processes. As research in metabolism has been ongoing for many years, the evolutionary models for metabolic pathways and networks have been developed [11], [12]. Both the retrograde and the pathchwork models attempt to explain the evolution of metabolic networks in response to adaptation phenomena. These models illustrate the metabolic network evolution either through changes in metabolite availability or due to enzyme specificity in response to gene duplication events [13]. By regulating the expression of genes involved in various metabolic processes, miRNAs can subsequently play a role in metabolic network restructuring and species evolution in general. The importance of tight regulation of miRNAs within the specific metabolic pathways (**Figure 2**) as well as their impact on metabolism alternation in the human lineage has not been previously described. Therefore, these results may potentially provide an insightful model for studying such miRNA-mediated regulatory interactions.

Equally important, is the fact that certain disease associated pathways are predicted to lose miRNA-dependent regulation in humans (**Figure 2**). The importance of this observation in the development of human diseases cannot be deduced from this work, however, the uniqueness of miRNA regulation in human linage of these pathways cannot be overlooked.

A large number of mammalian miRNA genes are known to be co-expressed with their host or neighboring genes [10]. Recent studies have demonstrated that such miRNAs can control the expression of their host genes by exerting synergistic and/or antagonistic regulatory effect. It was demonstrated that about 30% of human miRNAs are located within CNV regions of the genome[14]. Gene targets of these miRNAs have significant functional differences compared to the target genes of miRNAs hosted in non-CNV regions[15]. In this study we analyzed the number of CNV and non-CNV genes that host miRNAs in the eight different species and showed that the human genome has more miRNAs within CNV regions instead of non-CNV locations. On the contrary, in other species such as chimpanzee, macaque and rat, “host miRNAs are equally distributed between CNV and non-CNV regions. These data correlate with our previous observations where it was shown that human CNV genes are targeted by more miRNAs than chimpanzee CNV genes [3]. Combined, these results suggest a unique co-evolution of CNV-miRNAs in the human lineage which effectively augments the regulatory complexity and evolvability of the genomes.

Understanding the biological significance of these specialized miRNA-CNV genes interactions in the human genome is not a trivial matter. The complexity of the molecular and metabolic networks involving different genomic elements in humans, however, indicates a unique control of the genome plasticity in this lineage. Overrepresentation analysis of the binding sites of miRNAs within pathways and curated gene classes in human CNV regions may shed some light to the biological networks more tightly regulated by miRNAs. Our analysis highlights a specific regulation of cancer related genes in humans by miRNAs (**Table 6**) that might provide insights both to the unique changes in the molecular interactions in humans as well as to the evolution of the disease itself.

The co-evolution of CNVs, and miRNAs and their molecular interactions contributed to maintain a balance in gene expression during evolution. Understanding the unique characteristics of CNV-miRNA interactions in the human lineage can unravel the difference in the regulatory complexity and evolvability between humans and other species.

## Materials and Methods

### Acquisition of homologous CNV and non-CNV genes across 8 species

All known human CNV genes (n = 10,529) were downloaded from the Database of Genomic Variants (DGV at http://projects.tcag.ca/variation/downloads/)[16], [17]. The information on the homologous genes for 7 other species [chimpanzee (Pan troglodytes), macaque (Macaque Macaca), mouse (Mus muspculus), rat (Rattus norvegicus), chicken (Gallus gallus), dog (Canis lupus) and cow (Bos primigenius)] was collected from HomoloGene database (ftp://ftp.ncbi.nih.gov/pub/HomoloGene/build66/). Thereafter, a comprehensive atlas of homologous CNV and non-CNV genes was constructed by mapping the human CNV genes against the homologues genes of the 7 other species. A total of 9,337; 8,639; 9,543; 8,778; 7,139; 9,161 and 9,067 homologous CNV genes were observed (**Table 1**). All genes that are encoded in non-CNV regions were then downloaded from NCBI and assembled the non-CNV gene list. A total of 7,391; 9,796; 7,248; 6,761; 5,241; 7,051 and 7,113 non-CNV genes were observed (**Table 1**).

### miRNA binding site predictions within 3′-UTR of CNV genes across 8 species

The 3′-UTR sequence of CNV genes of 8 species were extracted from the ftp site of RefSeq release 53 (ftp://ftp.ncbi.nih.gov/refseq/). The mature sequence of human miRNAs (n = 1,921) was obtained from miRBase release 18 (ftp://mirbase.org/pub/mirbase/18/) [18]. An *in silico* screening for the possible binding sites between the downloaded sequences of CNV genes of 8 species and human miRNAs was accomplished by employing the miRWalk algorithm [19]. In parallel, the putative miRNA-target prediction datasets on these 8 species were also downloaded from TargetScan[20]. Integrating the prediction results from 2 or more prediction algorithms helps in reducing the number of putative targets and also helps in avoiding false positive predictions[21] [3], [22]. Therefore, the miRNA-target predictions only identified by both algorithms were considered for further analysis.

### Pathway enrichment analysis

The homologous CNV genes identified as the putative targets of human miRNAs with both algorithms (miRWalk and TargetScan) were selected. The total number of targets (CNV genes) for each human miRNAs and their families were calculated. The average means (total number of targets/820 miRNAs) for all known miRNAs and their families were then estimated for the 8 species. In a next step, the putative targets (CNV genes) were classified into two lists i.e. above and below average mean categories. Further, the putative targets of these 2 categories were used for identifying significantly enriched pathways by considering all KEGG pathways [23] with the help of a customized “*R script*” by implementing Fisher's exact test with the Benjamini and Hochberg (BH) as a multiple testing (background correction) method with 5% level of significance.

### Classification of homologous CNV genes into the curated classes

The information on datasets of different curated gene classes (such as cell differentiation, cytokines and growth factors, homeo-domain, transcription factors, etc) was gathered from GSEA (http://www.broadinstitute.org/gsea/) [24] and PubMed. The homologous genes (CNV and non-CNV) were then mapped and separated into these curated gene classes.

### Genomic location scanning of homologous genes of 8 species

The genomic coordinates of homologous genes (CNV and non-CNV) and known miRNAs of 8 species were downloaded from NCBI ftp site (ftp://ftp.ncbi.nlm.nih.gov/gene/DATA/) and miRBase release 18 [18], respectively. Afterwards, the genomic locations of homologous genes were scanned against the coordinates of known miRNAs using a customized “*Perl script*”.

### Overrepresentation analysis of miRNA binding sites within pathways, curated classes and miRNA host genes

A customized “*R script*” was utilized to perform an overrepresentation analysis (ORA) for identifying the significantly enriched binding sites of human miRNAs and their families within the representative members of significant pathways, curated classes and the host genes.
